# Keratotic Basal Cell Carcinoma of the Scalp: A Case Report of a Rare Histopathological Variant

**DOI:** 10.7759/cureus.116478

**Published:** 2026-09-18

**Authors:** Fahad AlSharhan, Reem AlQusaimi, Fawziah AlRujaib, Fatima Shamsuddin, Layal Khoursheed, Abdullah AlShatti, Qutov Alkhaldi, Dana Alobaid, Fay Alfayez, Fatma AlAmiri

**Affiliations:** 1 Dermatology, Sheikh Jaber Al-Ahmad Al-Sabah Hospital, Kuwait City, KWT; 2 Dermatology, Abdulkareem AlSaeed Dermatology Center, Kuwait City, KWT; 3 Pathology, Sheikh Jaber Al-Ahmad Al-Sabah Hospital, Kuwait City, KWT; 4 Medicine and Surgery, Sheikh Jaber Al-Ahmad Al-Sabah Hospital, Kuwait City, KWT; 5 Medicine, Sheikh Jaber Al-Ahmad Al-Sabah Hospital, Kuwait City, KWT; 6 General Surgery, Farwaniya Hospital, Kuwait City, KWT; 7 Medicine, Kuwait University, Kuwait City, KWT; 8 Medicine, Amiri Hospital, Kuwait City, KWT

**Keywords:** basal cell carcinoma, dermatopathology, keratinization, keratotic basal cell carcinoma, trichilemmal neoplasms

## Abstract

Basal cell carcinoma (BCC) is the most common cutaneous malignancy and demonstrates considerable histopathological diversity. Keratotic BCC is an uncommon variant characterized by prominent keratinization within otherwise typical BCC architecture, which may create diagnostic overlap with other keratinizing and follicular neoplasms.

We report a 51-year-old man with a longstanding scalp lesion and a history of prolonged occupational sun exposure. Histopathological examination demonstrated characteristic features of BCC with prominent keratinization and squamous differentiation. Given the follicular association and keratin-filled spaces, a trichilemmal neoplasm was considered; however, the overall morphology and supportive immunohistochemical findings established the diagnosis of keratotic BCC.

This case highlights an uncommon morphological variant of BCC and emphasizes the importance of recognizing keratotic differentiation within its histopathological spectrum. Increased awareness of this variant may facilitate accurate distinction from other keratinizing and follicular cutaneous neoplasms.

## Introduction

Basal cell carcinoma (BCC) is the most common cutaneous malignancy, arising predominantly on sun-exposed areas of the head and neck and strongly associated with ultraviolet (UV) radiation exposure. Although generally characterized by an indolent clinical course, BCC demonstrates substantial histopathological heterogeneity, with several recognized growth patterns and forms of differentiation. This morphological diversity has resulted in multiple histopathological classification systems and may occasionally complicate diagnostic interpretation [1].

Keratotic BCC is an uncommon morphological variant distinguished by prominent keratinizing differentiation within otherwise typical BCC architecture. Despite the high prevalence of BCC overall, keratotic BCC remains infrequently reported in the literature, with recent publications continuing to describe it as an uncommon presentation [2,3]. We present a longstanding keratotic BCC of the scalp in a 51-year-old man, with the aim of highlighting this unusual histopathological presentation and contributing to the limited literature documenting the morphological spectrum of this variant.

## Case presentation

A 51-year-old Kuwaiti man, previously healthy, presented with a 30-year history of a slowly enlarging scalp lesion. The lesion was first noticed approximately 30 years earlier while the patient was serving in the military, during which he reported prolonged daily occupational sun exposure. He initially described the lesion as a small, painless papule located over the central scalp. As it was asymptomatic, he did not seek medical attention at the time.

Over the subsequent three decades, the lesion gradually increased in size. Approximately three years prior to presentation, he began noticing intermittent yellowish discharge from the lesion that subsequently dried to form a persistent yellowish crust. More recently, the lesion demonstrated progressive enlargement with persistent crusting and discharge, accompanied by episodes of bleeding and ulceration, prompting him to seek medical attention. He denied the use of topical medications or other treatments for the lesion.

His medical history was unremarkable, with no known diabetes mellitus, hypertension, hyperlipidemia, or other medical or dermatological conditions. He was not taking any regular medications and had no known drug allergies. There was no family history of malignancy, including skin cancer. He denied constitutional symptoms, including unintentional weight loss, loss of appetite, or night sweats.

On physical examination, an 8 × 6 cm firm, adherent yellowish crust was noted over the midline scalp. The crust was softened with normal saline dressings and removed, revealing an underlying 4 × 6 cm flesh-colored, soft, raised lesion with an irregular surface and focal areas of bleeding. The surrounding skin appeared clinically normal. No palpable regional lymphadenopathy was identified (Figure 1).

**Figure 1:**
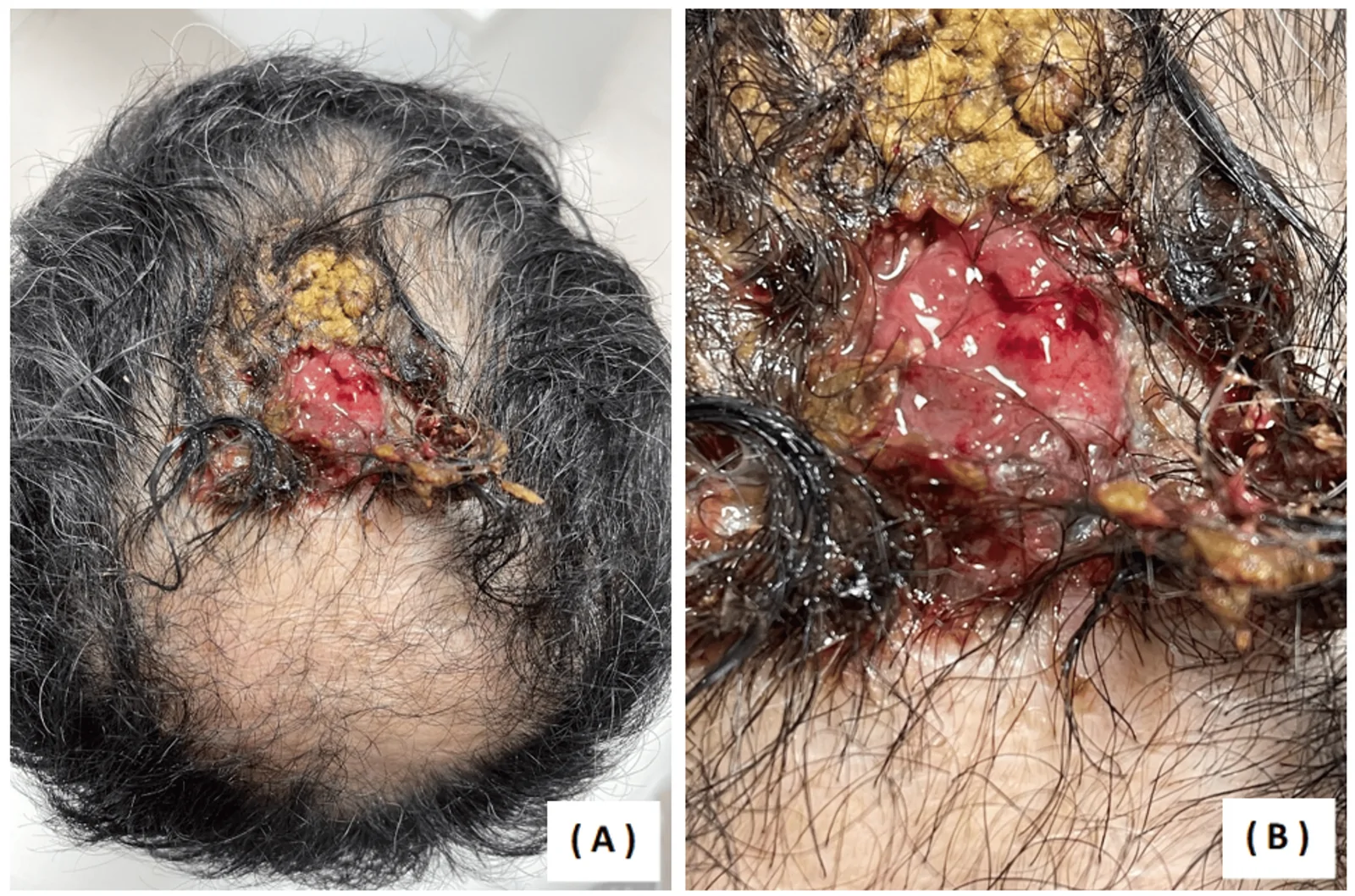
Clinical Presentation of the Scalp Lesion (A) Clinical appearance of the midline scalp lesion following partial removal of the overlying adherent yellowish crust. (B) Close-up view demonstrating the underlying flesh-colored, raised lesion with an irregular surface and focal areas of bleeding.

Based on the clinical appearance, prolonged course, and history of chronic sun exposure, the clinical differential diagnosis included BCC, squamous cell carcinoma (SCC), proliferating trichilemmal tumor, keratoacanthoma, and malignant adnexal neoplasms. A skin biopsy was subsequently performed to establish the diagnosis.

Histopathological examination of the scalp biopsy revealed an infiltrative dermal epithelial neoplasm composed of variably sized nests, lobules, and cords of basaloid cells. At low power, multiple tumor islands extended into the dermis and demonstrated prominent central keratinization, with keratin-filled cystic spaces. The tumor was associated with inflammatory and fibromyxoid stromal changes. On higher-power examination, the tumor cells exhibited hyperchromatic nuclei with scant cytoplasm and conspicuous peripheral palisading. Foci of squamous differentiation and prominent keratinization were identified within the basaloid tumor islands. Stromal retraction (clefting) surrounding the tumor nests, a characteristic feature of BCC, was also present. Focal epidermal ulceration with acute inflammatory exudate was noted. No lymphovascular or perineural invasion was identified (Figure 2).

**Figure 2:**
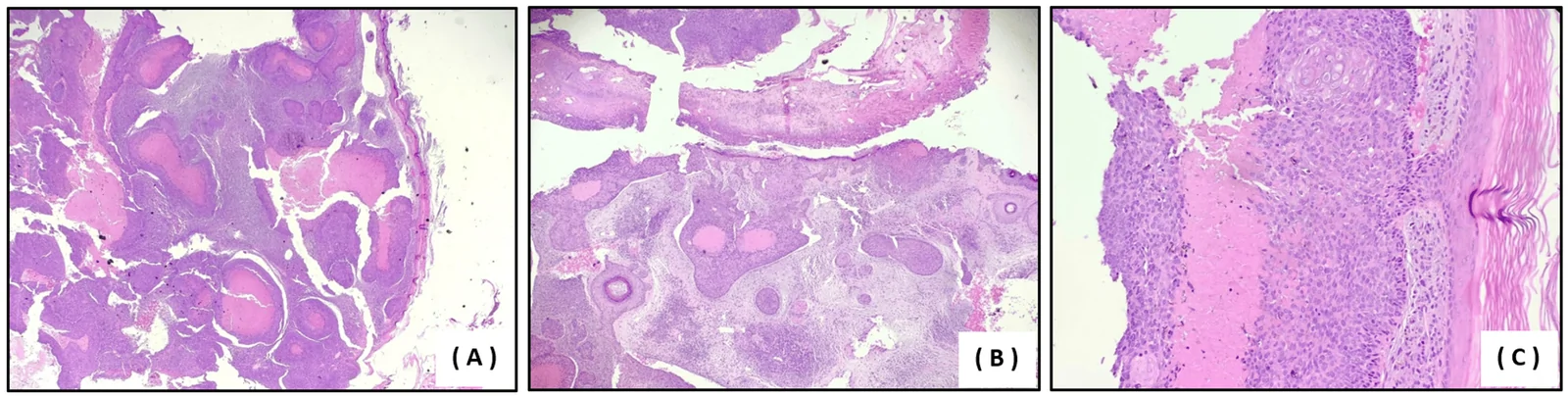
Histopathological Features of Keratotic Basal Cell Carcinoma Hematoxylin and eosin (H&E)-stained sections of the scalp lesion. (A) Low-power view showing an infiltrative proliferation of basaloid/squamoid tumor islands extending into the dermis, with prominent central keratinization (H&E, ×4). (B) Tumor islands demonstrating areas of central keratinization with surrounding desmoplastic and inflammatory stroma (H&E, ×4). (C) Higher-power view demonstrating basaloid cells with peripheral palisading, squamous differentiation, and prominent keratinization (H&E, ×20).

Immunohistochemical evaluation demonstrated strong, diffuse BerEP4 and B-cell lymphoma2 (BCL2) expression by the tumor cells, supporting basal cell differentiation. The neoplastic cells were negative for cluster of differentiation (CD)34, with staining confined to the surrounding stromal and vascular elements, and negative for epithelial membrane antigen (EMA), with staining limited to non-neoplastic elements (Figure 3). In view of the prominent keratinization and follicular association, a trichilemmal neoplasm was considered in the histopathological differential diagnosis. However, the overall morphology, particularly the basaloid architecture, peripheral palisading, and stromal retraction, together with the immunohistochemical profile, supported a final diagnosis of keratotic BCC. Following histopathological confirmation, the patient was referred to another center and surgical subspecialty for definitive surgical management. Details regarding subsequent excision, surgical margins, and clinical follow-up were not available.

**Figure 3:**
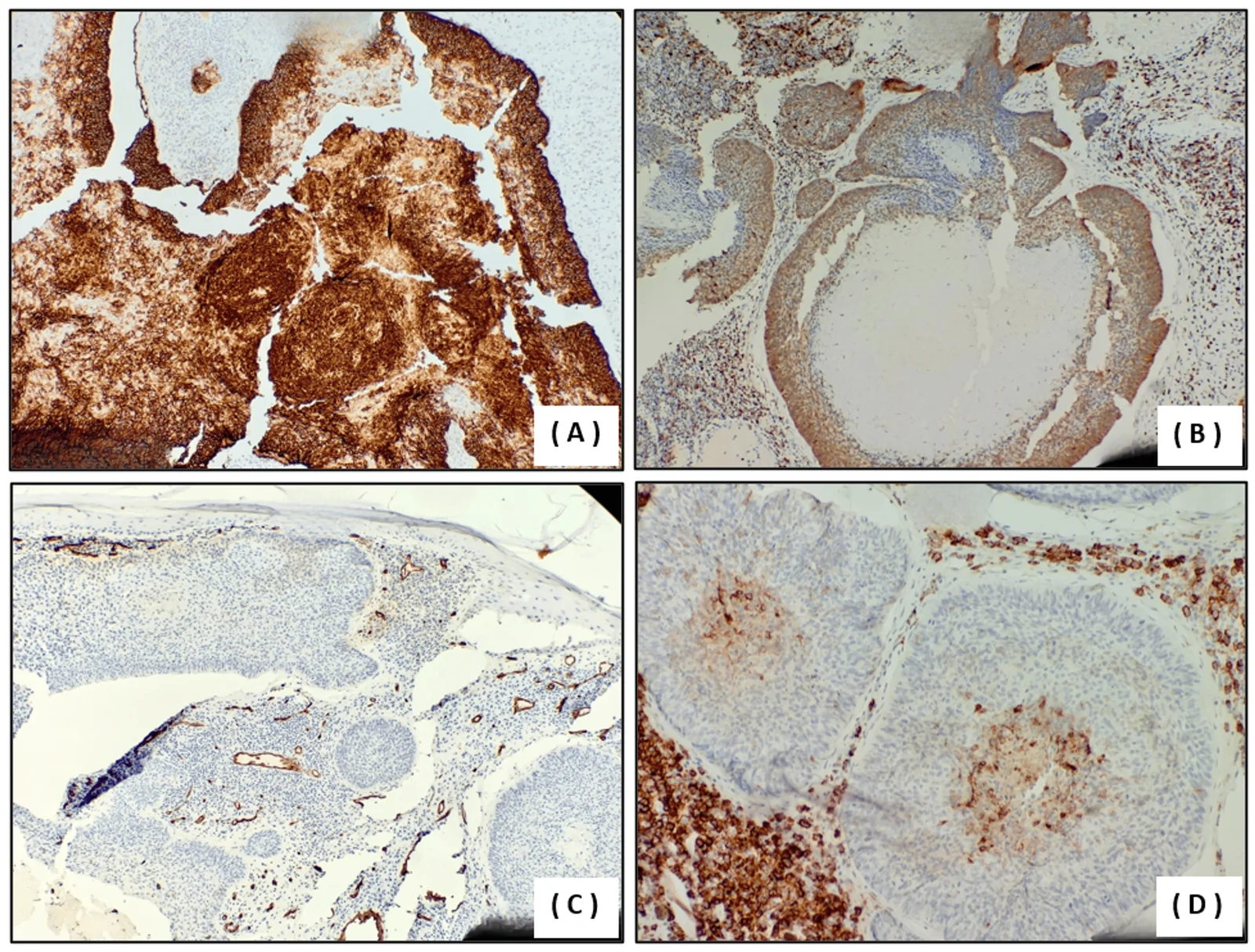
Immunohistochemical Profile of Keratotic Basal Cell Carcinoma Immunohistochemical staining of the scalp lesion. (A) The tumor cells demonstrate strong and diffuse positivity for BerEP4, supporting basal cell differentiation (BerEP4, ×10). (B) The tumor cells demonstrate diffuse BCL2 positivity, supporting a basal cell phenotype (BCL2, ×10). (C) CD34 highlights the stromal endothelial cells and vascular network, while the neoplastic cells are negative, demonstrating absence of significant CD34 expression by the tumor cells (CD34, ×10). (D) The tumor cells are negative for epithelial membrane antigen (EMA), with staining limited to non-neoplastic/entrapped epithelial and stromal elements (EMA, ×20).

## Discussion

BCC is the most common cutaneous malignancy and is strongly associated with cumulative and occupational UV exposure, particularly on the head and neck. Histopathologically, it demonstrates considerable morphological diversity, including follicular and keratinizing differentiation [1,4]. In the present case, the prolonged occupational sun exposure and scalp location are consistent with this established association.

Several classification systems have been proposed to categorize the diverse morphological patterns of BCC. Common patterns include nodular, superficial, micronodular, infiltrative, and morpheaform BCC, while mixed patterns may also occur [5]. Additional differentiation patterns include pigmented, adenoid, keratotic, infundibulocystic, and other adnexal forms [6]. Terminology varies across classification systems, reflecting the morphological diversity and imperfect reproducibility of BCC subtyping. Among these morphological patterns, keratotic BCC represents an uncommon variant characterized by otherwise typical features of BCC accompanied by prominent keratinization within the tumor islands [7].

Keratotic differentiation may include keratin-filled spaces or horn cyst formation within tumor islands and has been suggested to reflect infundibular or trichilemmal differentiation. Although reported at several anatomical sites, including the head and neck, this pattern remains infrequently encountered. Recognition is important because prominent keratinization may overlap histologically with other keratinizing and follicular neoplasms [8].

Histopathologically, our case demonstrated an infiltrative proliferation of basaloid tumor islands extending into the dermis, with peripheral palisading and characteristic stromal retraction. The most striking feature was prominent central keratinization, with keratin-filled spaces and foci of squamous differentiation within the tumor islands, supporting classification as the keratotic variant.

The histopathological differential diagnosis of keratotic BCC includes trichilemmal neoplasms, SCC, basosquamous carcinoma, and trichoepithelioma, all of which may demonstrate overlapping basaloid or keratinizing features [8,9]. Trichilemmal neoplasms may show follicular association and prominent keratinization; however, CD34 expression favors trichilemmal differentiation, whereas diffuse BerEP4 positivity supports BCC [10]. SCC may demonstrate prominent keratinization and squamous differentiation but typically lacks the diffuse BerEP4 expression characteristic of BCC, while EMA expression favors SCC. Basosquamous carcinoma demonstrates both basaloid and squamous differentiation and may show a transitional immunophenotype between BCC and SCC [11]. Trichoepithelioma may similarly contain basaloid nests and keratinous structures, creating substantial morphological overlap with keratotic BCC [8]. In our case, a trichilemmal neoplasm was particularly considered because of the keratin-filled spaces and follicular association; however, peripheral palisading, stromal retraction, diffuse BerEP4 and BCL2 positivity, and absence of CD34 and EMA expression supported keratotic BCC.

Immunohistochemistry further supported the diagnosis. Strong diffuse BerEP4 positivity and absent CD34 expression favored BCC over a trichilemmal neoplasm, while the BerEP4-positive/EMA-negative profile supported BCC over SCC [11]. Diffuse BCL2 expression was also consistent with a basal cell phenotype. Nevertheless, immunohistochemistry should remain adjunctive to the characteristic histopathological findings [10].

This case illustrates how prominent keratinization and follicular association may obscure the basal cell origin of the tumor and create diagnostic overlap with other keratinizing neoplasms. Recognition of the characteristic BCC architecture, supported by appropriate immunohistochemistry, is therefore important for accurate classification.

## Conclusions

Keratotic BCC represents a distinctive and uncommon expression within the broad morphological spectrum of BCC. This case adds to the limited literature documenting this variant and illustrates its occurrence as a longstanding, large scalp lesion with prominent keratinizing differentiation. Greater awareness and continued reporting of such uncommon variants may contribute to a better understanding of the morphological diversity of BCC and strengthen their recognition in routine dermatopathological practice.
